# Occurrence and Chemistry of Tropane Alkaloids in Foods, with a Focus on Sample Analysis Methods: A Review on Recent Trends and Technological Advances

**DOI:** 10.3390/foods11030407

**Published:** 2022-01-30

**Authors:** Lorena González-Gómez, Sonia Morante-Zarcero, Damián Pérez-Quintanilla, Isabel Sierra

**Affiliations:** Departamento de Tecnología Química y Ambiental, E.S.C.E.T, Universidad Rey Juan Carlos, 28933 Madrid, Spain; lorena.gonzalez@urjc.es (L.G.-G.); sonia.morante@urjc.es (S.M.-Z.); damian.perez@urjc.es (D.P.-Q.)

**Keywords:** tropane alkaloids, atropine, scopolamine, food sample preparation, solid–liquid extraction, solid-phase extraction, QuEChERS, new sorbents, molecularly imprinted polymers, mesostructured silica-based materials

## Abstract

Tropane alkaloids (TAs) are natural toxins produced by different plants, mainly from the Solanaceae family. The interest in TAs analysis is due to the serious cases of poisoning that are produced due to the presence of TA-producing plants in a variety of foods. For this reason, in recent years, different analytical methods have been reported for their control. However, the complexity of the matrices makes the sample preparation a critical step for this task. Therefore, this review has focused on (a) collecting the available data in relation to the occurrence of TAs in foods for human consumption and (b) providing the state of the art in food sample preparation (from 2015 to today). Regarding the different food categories, cereals and related products and teas and herbal teas have been the most analyzed. Solid–liquid extraction is still the technique most widely used for sample preparation, although other extraction and purification techniques such as solid-phase extraction or QuEChERS procedure, based on the use of sorbents for extract or clean-up step, are being applied since they allow cleaner extracts. On the other hand, new materials (molecularly imprinted polymers, mesostructured silica-based materials, metal–organic frameworks) are emerging as sorbents to develop effective extraction and purification methods that allow lower limits and matrix effects, being a future trend for the analysis of TAs.

## 1. Introduction

Tropane alkaloids (TAs) are secondary metabolites produced by various plant species, mainly belonging to the Solanaceae family, in addition to a variety of other families (Brassicaceae, Erythroxylaceae, Euphorbiaceae, Convulvulaceae) [1,2,3]. There are more than 200 different TAs identified, and they can be found anywhere in the plant, including seeds, fruits, flowers, leaves and stems [2,4]. The most studied TAs in foods are atropine (At) and scopolamine (Sc).

**Figure 1:**
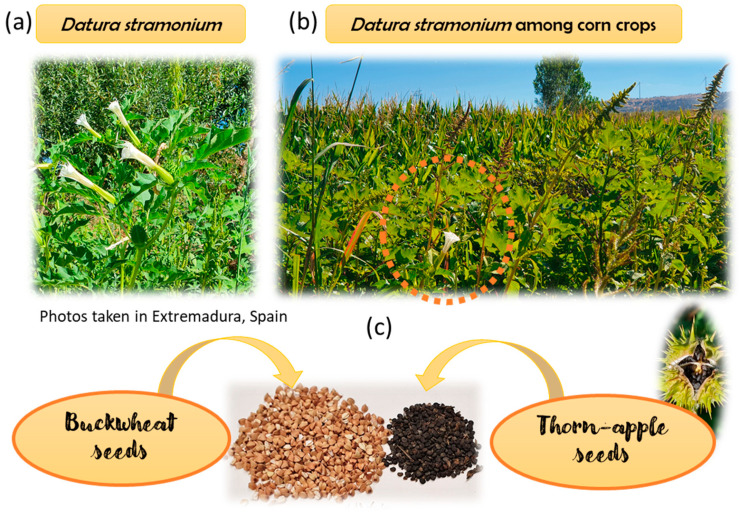
(**a**) *Datura stramonium* plant, (**b**) *Datura stramonium* among corn crops and (**c**) similarity of *Datura stramonium* seeds with buckwheat seeds.

Crop contamination by plants of the Solanaceae family is the most widespread source of accidental TAs consumption. Specifically, *Datura*, *Hyoscyamus* and *Atropa* species are the main ones responsible for food and feed products contamination by TAs. This is because they grow easily as weeds in crops of different plant-foods. In addition, these alkaloids are found in all parts of TA-producing plants, so cross-contamination especially with seeds but also with leaves, roots, fruits and flowers is frequent due to fast and mechanical harvesting. The seeds of *Datura stramonium* (jimson weed or thorn apple), widely distributed in all the warm regions of the world, along with other *Datura* spp., such as *D. ferox* are the ones that appear the most in foods [3]. *D. stramonium* produces numerous seeds that are encapsulated in a kind of apple-shaped fruit, hence its name thorn apple. These seeds have generally been found as impurities in numerous crops of linseed, soy, millet, sunflower and buckwheat [5] (Figure 1). For example, in Uganda in 2019, humanitarian food aid (product known as Super Cereal composed by maize and soya) contaminated with TAs of *D. stramonium* was responsible of the foodborne outbreak which caused over 300 hospitalizations and 5 deaths [6]. In this sense, it is necessary that food producers and manufacturing companies ensure, in the next years, the reduction of the amount of undesirable plants producing TAs in crops, raw materials and finished products, following good agricultural and manufacturing practices. In addition, the adequate application of food safety control measures (including the establishment of maximum limits) can aid in the reduction of TAs in foods [6].

The main objective of this review is to offer information about the occurrence of TAs in plant- and animal-derived foods, with a special interest in At and Sc; attend to regulatory aspects; and to summarize the most used sample treatment preparation protocols, including the use of new materials for analyzing TAs in food matrices. The bibliography was reviewed from 2015 to 2021, bearing in mind the latest reviews published.

## 2. Chemistry and Occurrence of TAs in Foods

### 2.1. Chemistry

**Figure 2:**
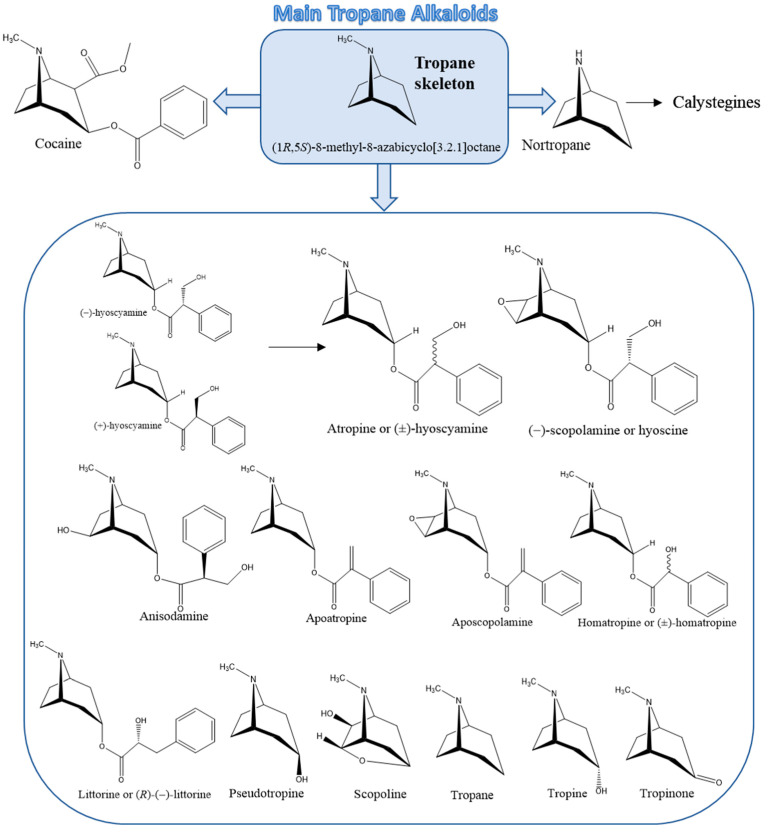
Common skeleton structure of tropane and principal tropane alkaloids.

TAs are a type of alkaloids with a tropane ring in their chemical structure, characterized by a two-ringed structure with pyrrolidine and piperidine rings sharing a single nitrogen atom and two carbon atoms (Figure 2). Most of them are esters derived from organic acids and hydroxytropanes [2]. The most important TAs in foods are At, which is a racemic mixture of (±)-hyoscyamine, and (−)-Sc or hyoscine. Although cocaine and calystegines have also a tropane ring (Figure 2), so they can be included in the TAs group, these compounds will not be discussed in this review. Cocaine and other coca alkaloids from *Erythoxylum coca* have no interest in food analysis, as they are used as a drug of abuse. Calystegines are recently discovered polyhydroxylated nortropane alkaloids. Due to their inability to pass the blood–brain barrier, these compounds do not show any psychoactive effects and, until now, their occurrence and analysis in food samples have received little research attention [7]. 

Despite all TAs having the same basic structure, they differ in their biological and pharmacological properties. At and Sc have been used throughout history in medicine, generally administered at low doses as drugs in the form of salts, such as atropine sulfate, or as semi-synthetic derivatives such as homatropine bromide or N-butylscopolamine bromide [1]. Their use has served to treat multiple symptoms and pathologies such as nausea, vomiting, heart or respiratory problems and also as an antispasmodic in gastrointestinal problems, as antiallergic drugs, as a treatment against organophosphate compounds and even as a pupil dilator for ophthalmic treatment [7,8,9,10]. However, and despite their great use, these compounds have been implicated in numerous intoxications due to the consumption of TA-containing plants or foods contaminated with these plants. Its toxic effects are due to the fact that they are anticholinergic compounds, which avoid the binding of acetylcholine with the muscarine receptor [1,4,8,11]. These toxic effects cause tachycardia, muscle spasms, mydriasis, delirium and sometimes can even cause death [2,8].

### 2.2. Occurrence

#### 2.2.1. Cereals, Pseudo-Cereals, Legumes and Grains

Contaminated cereals, pseudo-cereals, legumes and grains are of particular importance because these kinds of food-products are a major part of the diet for all age groups in the population. In relation to TAs’ occurrence, one of the most studied has been buckwheat (*Fagopyron esculentum* L.). In fact, this pseudo-cereal has caused great commercial interest for its healthy properties, being rich in polyphenols, vitamins and proteins [12]. Buckwheat flour and other gluten-free flours (i.e., amaranth, chickpea, pea, corn, rice, millet and quinoa flours, among others) are used as wheat flour substitutes in the production of gluten-free bread, cakes, cookies, pasta and snacks. These gluten-free foods have gained great popularity in the last years, not only in the celiac community, which emphasizes the need to carefully control the occurrence of TAs present in these products. This is of especial importance as the seeds of *Datura stramonium* are very similar in size and shape to those of buckwheat and other seed matrices such as linseed, soy, sorghum, and millet (Figure 1c). Additionally, the commercialization of organic foods is common so that the use of phytosanitary products is avoided, which results in the crops growing with a greater number of weeds. This can cause the contamination of the flours and derived products such as pasta, bread or baby foods, which are currently highly consumed.

Table 1 shows the occurrence of TAs in cereals, pseudo-cereals, legumes and grains. In some studies, high concentrations of At and Sc have been found, together with other alkaloids. For example, in the study of 26 buckwheat-derived organic foods carried out by Cirlini et al. [13], At and Sc were found in a sample of buckwheat flour (83.9 and 10.4 µg/kg, respectively) and in a sample of buckwheat pasta (21.3 and 5.7 µg/kg, respectively). In addition, positive samples in At were found by González-Gómez et al. [14] in buckwheat grains and flours in a range between 6.7 and 21 µg /kg. Other cereals, pseudo-cereals, legumes and grains are comparatively less studied. However, amounts that exceed the maximum level allowed for millet and sorghum according to the Commission Regulation (EU) 2021/1408 [15] have been reported. In that respect, Marín et al. [16] found 13 µg/kg of At and 23 µg/kg of Sc in a sample of millet flour, and in the study of González-Gómez et al. [14], a sorghum flour contained 15 µg/kg. In the same study, in a sample of teff flour, the sum of At and Sc was higher than 100 µg/kg, which evidenced that these kinds of grains and flours must also be carefully studied.

In cereal-based food for infants and young children, Mulder et al. [17] detected TAs in 22% of the 113 samples analyzed (breakfast cereals to be prepared with milk or water, biscuits and cookies). TAs were only detected in breakfast cereals to be prepared with milk (21 samples), and the mean levels (sum of At and Sc) were 4.6, 4.4 and 0.5 µg/kg for samples collected in 2011, 2012 and 2014, respectively (maximum 80.8 µg/kg in 2011). The lower levels found in 2014 were possibly due to measures taken by producers in response to the EFSA Opinion of 2013 [2]. However, in a more recent study, Marín-Sáez et al. [18] analyzed 18 cereal-based baby foods finding a positive sample of biscuits with 11.5 µg/kg of At and 2.8 µg/kg of Sc, which contained TAs levels above the EU regulatory limit of 1 µg/kg for each alkaloid. On the other hand, in a study of Baslé et al. [19] with samples from Asian and African countries, At and Sc were not detected in any of the four infant-cereals products analyzed, which were purchased in Singapore.

As it can be seen in Table S1, several alerts on the European Rapid Alert System for the Food and Feed (RASFF) portal [20] were issued from 2015 to 2021, in which different cereals and bakery products were found to be contaminated with At and Sc (69% of the notifications, Figure S1). The highest levels were reported for a sorghum flour with concentrations of At of 1200–1500 µg/kg and Sc of 360–460 µg/kg. On the other hand, millet and its derivatives were the products with the highest number of alerts (38%), followed by corn and its derivatives, with 33% of the alerts (Figure S1).

**Table 1:** Occurrence of TAs in cereals, pseudo-cereals, legumes and grains.

Foods (Nº of Samples Analyzed)	Nº of Samples with At (Range)	Nº of Samples with Sc (Range)	Other TAs	[Ref.]
Breakfast cereals, breakfast cereals with milk, biscuits, cookies (113 samples)	21 of breakfast cereals (0.09–65.6 µg/kg)	18 of breakfast cereals (0.28–15.2 µg/kg)	Anisodamine, aposcopolamine, homatropine, anidosine	[17]
Flours (buckwheat, millet and corn), cereal-based foods for children, breakfast cereals, biscuits and pastry, pasta and bread, legumes, stir-fry mixes and oilseeds (1305 samples)	46 of flours (0.5–149 µg/kg), 42 of cereal-based foods for children (0.5–3.73 µg/kg), 15 of breakfast cereals (0.5–90.83 µg/kg), 24 of biscuits and pastry (0.5–1.85 µg/kg), 18 of bread (0.5–3.80 µg/kg), 20 of legumes, stir-fry mixes and oilseeds (0.5–0.11 µg/kg)	46 of flours (0.5–198.5 µg/kg), 42 of cereal-based foods for children (0.5–1.86 µg/kg), 15 of breakfast cereals (0.5–17.64 µg/kg), 24 of biscuits and pastry (0.5–0.65 µg/kg), 18 of bread (0.5–0.36 µg/kg), 20 of legumes, stir-fry mixes and oilseeds (0.5–0.09 µg/kg)	6-HO-tropinone, nortropinone, pseudotropine, scopine, scopoline, tropine, tropinone, convolamine, convolidine, convolvine, fillalbine, anisodamine, apoatropine, aposcopolamine, homatropine, littorine, noratropine, norscopolamine	[21]
Buckwheat, buckwheat flour and pasta; soy and soy flour; peeled millet and millet flour; linseed and linseed flour (15 samples)	1 of buckwheat (<1 µg/kg) 1 of millet flour (13 µg/kg)	1 of buckwheat (<2 µg/kg) 1 of millet flour (23 µg/kg)	Anidosamine, littorine, tropinone	[16]
Buckwheat, buckwheat flour and pasta, soy, wheat, amaranthus grain, chia seeds, peeled millet (8 samples)	N.D	N.D	Three scopolamine transformation products	[22]
Buckwheat flour, pasta and bakery (26 samples)	1 of flour (83.9 µg/kg) 1 of pasta (21.3 µg/kg) 1 of bakery (13.9 µg/kg)	1 of flour (10.4 µg/kg) 1 of pasta (5.7 µg/kg)	N.St	[13]
Cereal based baby foods (pap, biscuits, snacks and grissines) (18 samples)	1 of biscuits (11.5 µg/kg)	1 of biscuits (2.8 µg/kg)	Anidosamine, homatropine, apoatropina	[18]
Bread (wheat, multi-grain, rye, wheat-rye) (40 samples)	N.D	1 of wheat-rye bread (0.22 µg/kg)	N.D	[23]
Wheat, corn, rice, oat and millet flours, mixed cereals flours, infant cereals, cereal-based products (95 samples)	1 of tomato rice flakes product (9.6 µg/kg)	1 of tomato rice flakes product (2.6 µg/kg)	N.St	[19]
Buckwheat and buckwheat flour, quinoa, amaranth, teff flour, refined corn flour, corn flour, blue corn flour, sorghum flour, peeled millet, green and red lentil flours, chickpea flour, pea flour (15 samples)	3 of buckwheat grain and flour (6.7–21 µg/kg), 1 of quinoa (7.1 µg/kg), 1 of teff flour (78 µg/kg), 1 of refined corn flour (7 µg/kg), 1 of sorghum flour (15 µg/kg), 1 of peeled millet (6.9 µg/kg)	1 of teff flour (28 µg/kg)	N.St	[14]

Abbreviations: At: atropine. N.D: not detected or below the limit of detection. N. St: not studied. Sc: scopolamine. TAs: tropane alkaloids.

#### 2.2.2. Teas, Herbal Teas, Herbs and Spices

Plant-derived ingredients are widely consumed worldwide for a multitude of medicinal and culinary purposes. They improve the sensory properties of foods and help to extend their shelf life, so they are frequently used as food condiments in cuisine and as natural additives in the food industry. In addition, some of them are also valuable as ingredients to prepare beverages (teas and herbal teas), herbal extracts and supplements with a variety of uses. Teas, herbal teas, herbs and spices can also be accidentally contaminated with seeds and leaves of TA-producing plants. Contamination most likely occurs during harvest or processing. Teas and herbal tea infusions positive for TAs can pose a serious health problem, as these toxins are relatively heat-stable, and some heavily contaminated products have been found in the market, so they can contribute significantly to the population’s exposure to TAs. In this sense, different poisoning cases caused by the contamination of herbal teas have been reported [24], and RASFF notifications from 2015 (Table S1) showed that seven alerts were found to be in teas and herbal teas (from 26.8 to 543.1 µg/kg of At and from 23 to 488.7 µg/kg of Sc). On the other hand, the current RASFF notification has evidenced that other botanical ingredients may also contain high amounts of these natural toxins. In this sense, an RASFF alert in 2018 deserves to be mentioned, due to the high TAs concentration found in whole cumin seeds from Hungary with 16178 µg/kg for At and 4658 µg/kg for Sc (Table S1 and Figure S1, see Supplementary Materials). The positive findings of TAs in these products warrant a more extensive survey to determine the extent of TAs contamination in this type of product.

As can be seen in Table 2, the occurrence of TAs has been mainly studied in tea (black, green, red) but also in other herbal teas such as peppermint, chamomile and rooibos, among others [25,26,27]. In some of these products, positive samples exceeded the 25 µg/kg of TAs (as the sum of At and Sc) established as maximum level by the Commission Regulation (EU) 2021/1408 in dried products for herbal infusions (50 µg/kg in anise seeds) [15]. At and Sc were the most prominent TAs found, but others such as homatropine, anidosamine, apoatropine, physoperuvine, pseudotropine and tropine were also detected [25,26]. In 2015, Shimshoni et al. [27] investigated the occurrence of TAs in herbal teas and teas. The highest amount of At and Sc was found in peppermint tea (181 µg/kg, mean total At and Sc). In a sample of rooibos tea and chamomile, Sc was found at low levels (around 2 µg/kg). No TAs were detected in all black tea, green tea and melissa tea samples analyzed. Furthermore, fennel herbal tea samples were also mostly free of TAs, except for one sample containing 83 µg/kg of At and 11 µg/kg of Sc. In the study of Mulder et al. [21], where 121 herbal tea and tea samples purchased in Europe were analyzed, 70% of the samples contained TAs, At and Sc being the most frequent TAs, with a maximum amount of 429 µg/kg (sum of At and Sc) found in a sample of mixed herbal tea. In addition, several TAs were detected in 6 of the 11 tea and herbal tea samples evaluated by Romera-Torres et al. [26], with concentrations from 5 µg/kg (apoatropine) to 4340 µg/kg (sum of pseudotropine, physoperunive and tropine). On the contrary, in another study where 60 samples of herbal teas and herbal supplements (extracts and tablets) from the Italian market were analyzed, only 8% were found to be contaminated, mainly by the presence of At (69 µg/kg in an infusion of 32% *Taraxacum officinalis*, 22% *Curcuma longa*, chamomile and fennel seeds). More recently, in 16 samples of commercial culinary aromatic herbs (dried leaves of thyme, basil and coriander), González-Gómez et al. [28] found At in 14 samples analyzed (over an interval of <5–42 µg/kg) and Sc in 3 samples (between <5–34 µg/kg). Given the toxicity of At and Sc and the high concentrations that can be found in these types of products, the analysis of aromatic herbs and spices is highly convenient to control the maximum levels set for both toxins in order to ensure a safe consumption of these types of products.

#### 2.2.3. Other Plant-Based Foods

Vegetables and other plant-based food products can also be contaminated with TAs. This is because the leaves of these vegetables, for example, spinach, can be mixed with leaves from TA-producing plants such as *Datura* spp. In addition, despite boiling being a common cooking option for these products, boiling water may not be able to effectively extract TAs. Thus, in a recent work, Castilla-Fernández et al. [29] selected spinach products because of poisoning incidents caused by contamination with *D. innoxia* leaves owing to their similarity. The results of this study evidenced the presence of At and Sc in 16 out of the 66 products analyzed, including frozen spinach and spinach-based infant food products. At was found in the range of 0.02 to 4.52 µg/kg and Sc from 0.04 to 8.19 µg/kg. The Sc/At ratio in the analyzed samples evidenced contamination with *D. innoxia* as Sc was found in most cases at a higher concentration than At. These authors emphasize the need to enforce TA limits in other food products, such as spinach, as the lack of regulation in some food commodities might be a source of health issues, especially if these products are used in homemade infant food. In this sense, in a recent RASFF alert (Table S1, Figure S1) in deep frozen spinach puree from Slovakia, 1938 and 1164 µg/kg of At and Sc were found, respectively.

#### 2.2.4. Animal-Derived Foods

Very little work has been carried out examining the potential presence of TAs in animal-derived foods. In 2013, the EFSA concluded that the risk of TAs poisoning through the consumption of food of animal origin was unlikely [2]. This is because cattle generally do not consume plants that contain TAs, since they tend to have an unpleasant taste that makes animals avoid it. Only when the amount of available grass is restricted are cattle likely to consume these plants [3]. Therefore, exposure to these toxins is usually mainly through feed contaminated with TAs or through waters contaminated with TA drugs that end up in rivers and are consumed by animals [30]. In a recent study by Lamp et al. [31], the transfer of TAs from feed to milk was demonstrated in cattle for the first time. These authors conclude that since the mixing of various raw milk leads to a dilution of possible TAs, the occurrence of dairy products contaminated in the market can most probably be disregarded, but the situation might be different when raw milk is sold on the farm. In this regard, the analysis of TAs content in milk and other animal-derived foods (such as meat, eggs), other than feed, could help estimate current TAs exposure levels through the food of animal origin. As it can be seen in Table 3, from 2015, there is only a single paper dealing specifically with the determination of TAs in this kind of sample. In this work [30], the developed analytical method was applied to the analysis of TAs in 30 samples of porcine muscle, chicken eggs and whole and low-fat milk (10 of each matrix) obtained from different local markets in the Republic of Korea. None of these foods were contaminated by these compounds. Despite this, there is a need for more research on this type of food, to ensure that they are not a risk for human consumption.

Honey is a natural animal-derived foodstuff produced by bees. This product is of great interest nowadays due to its bioactive compounds with positive health effects. However, it is well-known that honey may contain different contaminants, including natural toxins. As consequence, some studies have evaluated the presence of TAs in honey (Table 3). For example, Martinello et al. [5] analyzed 40 honey samples purchased in local markets in Italy (multifloral and acacia honey). At was found in nine samples (22%), and five were found with concentrations ranging from 1.4 to 3.8 µg/kg, whereas Sc was never observed. In another study [32], 11 of the 19 samples analyzed were found positive in different TAs, but at trace levels, with the exception of a multifloral honey with 27 µg/kg of Sc. Instead, Thompson et al. (2020) [33] found low concentrations of TAs in 23 samples of honey analyzed. A type of honey from Greece (from thyme and wild flora) contained the highest concentrations, 0.012 µg/kg of At and 0.012 µg/kg Sc. Due to the small number of studies that have analyzed this food, it is difficult to draw any conclusions regarding the presence of TAs in honey.

## 3. Regulatory Aspects and Safety Issues in Food

As reported above, TAs are undesirable substances in food and feed. Because At and Sc exhibits anticholinergic activity, the European Food Safety Authority (EFSA) in 2013 established an acute reference dose (ARfD) of 0.016 µg/kg body weight expressed as the sum of (–)-hyoscyamine and (–)-scopolamine and recommended compiling analytical data on the occurrence of TAs in cereals and oilseeds [2]. As a result, in 2015, the European Commission recommended the control of TAs, at least At and Sc, in susceptible food such as gluten-free products, food supplements, teas and herbal infusions, legume, oilseeds, derived products of these and, especially, cereals and derived products (buckwheat, sorghum, millet, corn, and the flours of these), processed cereal-based foods for infants and young children, breakfast cereals, grain milling products and grains for human consumption [34]. In that respect, maximum levels for At and Sc were established by Commission Regulation (EU) 2016/239 in processed cereal-based foods and baby foods for infants and young children, containing millet, sorghum, buckwheat or their derived products (1 µg/kg of each one) [35]. Subsequently, a study carried out by the EFSA published in 2016 [21], in which 1305 samples of food of plant origin produced in Europe were analyzed, evidenced high concentrations of TAs in different types of foods such as flours, cereal-based foods, teas, legumes, etc. Two years later, in 2018, the EFSA published a scientific report on the assessment of acute dietary exposure to TAs in the population of the EU, taking into account the new occurrence data [36]. The results indicated that the ARfD was exceeded in different population groups, for several acute exposure estimates, which makes the presence of TAs a health concern. As a result, in August 2021, the European Commission extended the legislation to processed cereal-based foods and baby foods for infants and young children containing maize (1 µg/kg of At and 1 µg/kg of Sc) [15]. In addition, the maximum levels for the sum of At and Sc have been set for unprocessed or processed millet, sorghum, maize, maize for popping and buckwheat (5–15 µg/kg). In other products, such as some foodstuffs found to contain a high concentration of TAs and contributing significantly to the exposure of the population, as herbal infusions, maximum levels have also been established (25–50 µg/kg in dried products and 0.2 µg/kg in liquid products) [15].

**Figure 3:**
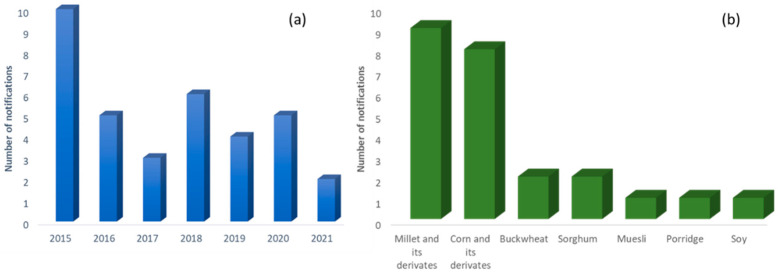
(**a**) Total number of notifications per year on the RAFSS portal and (**b**) number of notifications in the “cereals and bakery products” category from 2015 to 2021.

**Figure 4:**
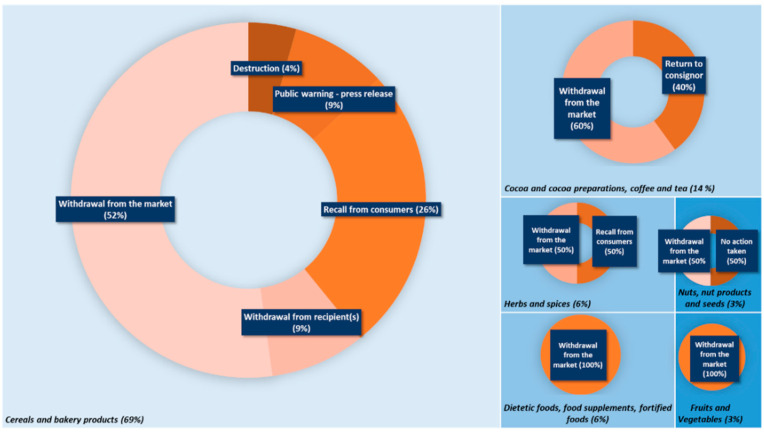
RASFF notifications of TAs between 2015 and 2021 by food category and action taken.

On the other hand, as it can be seen in Figure 3a, from 2015 to today, 35 notifications on TAs have been reported on the RASFF portal [20], 24 of them being in the category of the “cereals and bakery products” (Figure 3b). Eight different countries, Austria, Croatia, Czech Republic, France, Germany, Hungary, the Netherlands and the United Kingdom, have reported the appearance of At and Sc in different foods. Some of these products are imported from different European countries (see Table S1 in Supplementary Materials). The highest number of notifications were seen in the “cereals and bakery products” category, with 69% of the notifications; followed by the “cocoa and cocoa preparations, coffee and tea” category with 14% of the notifications; the “dietetic foods, foods supplements and fortified foods” and “herbs and spices” categories with 6% of notifications; and, finally, the “nuts, nuts products and seeds” and “fruits and vegetables” with 3%. The main action taken after the notification, in all the food categories, was withdrawal from the market (Figure 4).

Focusing on the RASFF data, it deserves to be mentioned the alert in deep frozen spinach puree contaminated with TAs of *D. stramonium* (more than 3000 µg/kg as sum of At and Sc) responsible for the foodborne outbreak that occurred in March 2021 in Slovakia and the Czech Republic with over 100 hospitalizations (Table S1). In this sense, it is clear the need to establish of a maximum level of TAs in other food products not included in the new Commission Regulation (EU) 2021/1408 [15]. Therefore, considering the high number of alerts and the toxic effects of these natural toxins for humans, continuous investigations to evaluate the occurrence in highly consumed plant- and animal-derived foods and to develop appropriate analytical methods for constant quality control are necessary to protect human health.

## 4. Sample Preparation and Analysis Methods of TAs in Foods

### 4.1. Chromatographic Analysis Conditions

As indicated in Section 2, some papers are available in relation to the occurrence of TAs in different food commodities for human consumption. However, for this task, the development of appropriate analytical methods is of great importance for the determination of these compounds, including extraction and clean-up steps, separation by chromatographic techniques and detection with mass spectrometry (MS). In this sense, the newly developed methods, as proposed by the European Commission, should preferably use high-performance liquid chromatography coupled to mass spectrometry (HPLC-MS) or gas chromatography coupled to mass spectrometry (GC-MS) in the case that HPLC-MS is not available [34]. The optimized and validated methods must reach quantification limits of less than 5 µg/kg for agricultural products, ingredients, food supplements and infusions; 2 µg/kg for final products; and 1 µg/kg for baby food according to the Commission Recommendation (EU) 2015/976 [34]. These limits refer to At (racemic mixture of (±)-hyoscyamine), since the separation of enantiomers is not always possible, and Sc.

Nowadays, HPLC is the most common technique used for the separation and quantitation of TAs. It was firstly reported in 1973 to separate At, Sc, homatropine and apoatropine [37]. Usually, TAs are analyzed in reverse phase mode with conventional C18 stationary phases [13,14,17,18,19,21,22,23,25,27,28,29,30,38,39,40], although C8 [5] and HILIC columns have also been used [16,26,32,33,41]. The mobile phase commonly consisted of a mixture of organic phases (methanol, acetonitrile or mixtures of them, sometimes acidified) and aqueous phases (water with different additives such as acetic and formic acids and salts such as ammonium hydroxide, ammonium formate or ammonium acetate). Regarding detectors, MS detectors predominate, considering that the lack of a strong chromophore in TAs molecules requires detections at low wavelengths, making it difficult to identify these compounds with UV detectors. For this reason, different analyzers based on MS are used to determine TAs, usually triple quadrupole (QqQ) [13,14,17,18,19,21,22,23,25,28,29,30,33,41,42] but also quadrupole-ion trap (QTRAP) [27,38]. High-resolution mass spectrometry analyzers (HRMS) such as the Orbitrap analyzer [5,16,18,26,32,39,40] or its variant quadrupole-orbitrap (Q-Orbitrap) [5] have also been used. Details of chromatographic conditions and validation parameters are shown in Table S2 (Supplementary Materials).

### 4.2. Sample Preparation for TAs Analysis in Foods

Sample preparation is the most important step in the analysis, especially with complex food samples, and is usually considered a bottleneck of analytical processes [43]. The objective of this stage is to reduce the interferences generated by the food matrix and improve the analyte signal in the instrument, since the matrix effect seriously affects the selectivity and sensitivity of the method. Additionally, there has been a tendency in the last years to develop new extraction and clean-up approaches by minimizing the number of steps to reduce both time and sources of error, moving towards more environmentally friendly techniques and improving the extraction efficiency and selectivity with the application of new advanced materials.

There are different alternatives that can be applied to sample preparation for TAs analysis (Table 4, Table 5 and Table 6). The choice of extraction and clean-up process, together with the selection of solvents and sorbents, generally depends on the type of alkaloid analyzed. Almost all TAs are soluble in water, being even more soluble at acidic pH, with the exception of apoatropine [16,37]. The most commonly used techniques for sample preparation are solid–liquid (SLE) and liquid–liquid extraction (LLE), including filtration and/or dilution steps. However, other alternative techniques based on extraction with sorbent such as solid-phase extraction (SPE) or in purification with sorbents for clean-up step such as QuEChERS (quick, easy, cheap, effective, rugged and safe) procedure have also been applied for TAs analysis in foods (Table 4, Table 5 and Table 6). In addition, aiming to improve the extraction performance of sample preparation techniques, some new materials have been developed and evaluated for TA analysis. This opens up a huge research window for the years ahead.

#### 4.2.1. Methodologies with Solid–Liquid and Liquid–Liquid Extraction Protocols

SLE is the most used technique to extract the TAs from the samples. This technique consists of extracting the compounds by contacting the solid sample with a solvent or mixture of solvents. Some parameters that can be varied in this extraction protocol include the time of extraction (or number of cycles), the solvent’s nature, the solvent pH and the sample:solvent ratio. The optimization of these parameters is of great importance to increase the extraction efficiency and to avoid TAs degradation.

As can be seen in Table 4, there are many studies that use this technique to extract TAs from different foods [13,17,23,25,27,33,38,39,40,41,42]. Table S2 (see Supplementary Materials) shows useful information about instrumental analysis and details about the validation parameters of these methods. Typically, mixtures of polar organic solvents or mixtures of acidic aqueous solutions with polar organic solvents are used for this task. For example, a mixture of methanol/water/formic acid (60/40/0.4, *v*/*v*/*v*) was used to extract six TAS from different samples of cereal-based products before HPLC-MS/MS analysis [17]. This solvent mixture was also tested in other works applied to spices, cereals and bread, obtaining similar recoveries for At and Sc [23,38]. Another mixture of methanol/water (2:1, *v*/*v*) with 0.5% formic acid was tested to extract 17 TAs from bread [39]. Cirlini et al. evaluated different mixtures of methanol/water, acetonitrile/water and both mixtures with a percentage of acid for SLE of At and Sc from buckwheat flour, buckwheat pasta and buckwheat bakery samples [13]. The methanol/water (3:2, *v*/*v*) combination acidified with 0.2% formic acid and 0.2% acetonitrile showed the best recoveries, ranging from 83–103% for Sc and from 78–102% for At. The matrix effect (ME) observed for At and Sc was different according to each matrix, pointing the necessity of a matrix-matched calibration for each category of commercial products. In pasta samples, important signal enhancement (ME: 133 and 144% for At an Sc, respectively) was estimated. In herbal teas, infusions and extracts, a mixture of acetonitrile/water (3:2, *v*/*v*) with 0.2% formic acid was used to extract 4 TAs with good recoveries, between 83 and 105% for all analytes [25]. However, in these types of samples, acetic acid/methanol (1:2, *v*/*v*) mixtures have also been tested with satisfactory recoveries [27].

On the other hand, a simple and effective salting-out assisted LLE was used by Thompson [33] for the determination of At and Sc in honey. Firstly, a concentrate aqueous solution of sodium acetate was used to dissolve the honey samples. Then, acetonitrile was added, but due to the high ionic strength of the aqueous solution, it was not miscible with water, and therefore, it was possible to perform a simple liquid–liquid partitioning procedure. The developed extraction protocol showed some benefits over with the QuEChERS procedure showing good recoveries ranging from 87 to 106%.

Although classical SLE and LLE methods are simple in general, they consume large amounts of solvents, and the extraction can be a long process. Table 4 shows that volumes of solvent used ranged from 10 to 40 mL (1 to 5 g of sample), and long periods of stirring (10 to 90 min) are necessary to extract the analytes. Moreover, the determination of these natural toxins in food samples is subjected to multiple matrix interferences that hinder their extraction and detection because of the high complexity of food samples. In this sense, some studies have confirmed that higher limits and lower matrix effects can be achieved by including a purification step. Accordingly, a suitable clean-up procedure of the sample extract before its instrumental analysis is important to achieve sensitive results and good analytical performance.

#### 4.2.2. Methodologies with Other Extraction and Purification Techniques

Despite the fact that methods for TAs analysis can be developed without a clean-up step (Table 4), in order to simplify the analytical procedure in routine analysis, it must be taken into account that food samples are very complex matrices, so clean extracts are usually required before the chromatographic analysis to improve sensitivity and selectivity. In addition, when MS detectors are used, it is not convenient to directly inject the sample extracts without a clean-up step, as it can foul the ionization source and decrease the sensitivity of the equipment, leading to more frequent, thorough and expensive maintenance of the detector. For TAs analysis, the SPE and QuEChERS procedures are the most applied techniques to obtain clean extracts. In addition, following actual tendency, new sorbent materials have been applied to develop improved sample treatment methodologies, mainly molecularly imprinted polymers (MIPs) or ordered mesostructured silicas (OMSs), among others. Table S2 (see Supplementary Materials) shows useful information about instrumental analysis and details about the validation parameters of these methods.

##### Solid-Phase Extraction

SPE is widely used in sample preparation because it is a preconcentration and/or purification technique. The SPE is based on the loading of an extract (of a liquid sample, for example, an infusion) onto a solid phase (sorbent) that is usually compacted in a cartridge or a syringe barrel, but disk-shaped supports are also available [44]. In SPE, the sorbent is responsible for the retaining of the target analyte, which after the interfering compounds of the sample extract are washed away, is eluted with an appropriate solvent. Different types of sorbents are available for SPE, based on organic polymers, silica or carbon. These sorbents can be modified with functional groups that provide different modes of interaction with the analytes (Van der Waals, hydrogen bonding, dipole–dipole or ion exchange), which is important when selecting the right sorbent.

Table 5 collects works that apply SPE in sample preparation to determine TAs in foods [16,18,21,26,40]. In general, mixed mode polymeric sorbents with anionic functional groups have been used to extract the basic TAs in acidic solutions (i.e., Oasis MCX^®^, Strata-X-C^®^). Specifically, the commercial Strata-X-C^®^ cartridge evaluated for this task contains a polymeric sorbent chemically modified with polar and strong cation exchange (SCX) groups [16,18,26,40]. In addition to the strong cation exchange interactions, this sorbent offers other retention mechanisms such as hydrophobic, hydrogen bonding and π–π [45]. Thanks to the strong ionic exchange retention mechanism, the sorbent can be washed with strong solvents (usually methanol with a low percentage of water) to remove neutral and anionic interferences. Finally, the elution of the target TAs is achieved with a mixture of methanol with ammonia (Table 5).

In this regard, Marín-Saéz et al. [16] and Romera-Torres et al. [26] compared different protocols for sample treatment, the application of the SPE being the one that offered the highest recoveries and allowed to increase the sensitivity. Furthermore, in both works, two SPE cartridges were compared, Oasis MCX^®^ and Strata X-C^®^. Oasis MCX^®^ showed lower recoveries compared to Strata X-C^®^ for the simultaneous determination of 13 TAs in cereals, pseudo-cereals, legumes, grains, teas and herbal teas. In addition, Strata-X-C^®^ have been used in online SPE, after a SLE procedure of cereal-based baby foods for the analysis or At and Sc, besides 11 other TAs [18]. The online system was directly coupled to different analyzers (QqQ and Orbitrap) (see Table S2). These authors conclude that with the developed methodology the analysis time can be minimized by increasing sample throughput in relation to offline approaches. Despite this, for both analyzers signal suppression was observed (ME ranges from −33 to −67%).

##### QuEChERS Procedure

The QuEChERS procedure is an appropriate approach as it involves simultaneous extraction and clean-up of samples for the determination of multiple analytes at the same time. The QuEChERS was developed in 2003 by Anastassiades et al. [46] as a green, user-friendly, quick and cheap useful procedure to perform multi-residue extraction of more than 200 pesticides from fruits and vegetables. Nevertheless, in the last years the QuEChERS concept has spread beyond its original field of application to be adapted to other analytes and food matrices.

In the original QuEChERS strategy, primary secondary amine (PSA) was used as dispersive clean-up sorbent to remove polar organic acids, polar pigments, some sugars and fatty acids due to its weak anion exchange properties. However, PSA is sometimes not capable of removing excessive interferences in complex matrices. For this reason, over the years, the QuEChERS method has been modified by the introduction of other clean-up sorbents, mainly graphitized carbon black (GCB) and octadecylsilane (C18), which are usually used in combination with PSA. Currently, QuEChERS have been applied for the determination of TAs in different foods (Table 6) [5,19,22,29,30,32]. 

As it can be seen in Table 6, some works applied the original QuEChERS protocol in cereals and spinach-based products with good recoveries between 87 and 107% [19,29]. Chen et al. [22] used a modified QuEChERS to determine At and Sc in buckwheat and related products. The protocol consisted of water and acetonitrile, containing 1% of acetic acid, for the extraction step, followed by a clean-up step with PSA and GBC. Both sorbents are highly used in samples of plant origin since they remove organic acids and polar pigments such as chlorophyll. Good recoveries (from 75 to 92%, except for chia samples) were observed. However, when the same modified QuEChERS procedure was used with teas and herbal tea samples [26], unsuitable recoveries were obtained, always lower than 60% (Table 6), compared with the use of SLE and SPE with Strata-X-C cartridges (Table 5).

In honey, different QuEChERS protocols have been used by Martinello et al. [5] and Romera-Torres et al. [32], with recoveries that ranged between 92 and 115% in the first study and between 71 and120% in the second. In order to achieve higher recoveries and a reduce matrix effect, Martinello et al. [5] also tested different extraction procedures with SPE Discovery^®^ DSC-SCX and Strata^TM^-X-C, but the results were not satisfactory compared to QuEChERS procedure. The validated method based on the QuEChERS protocol and HRSM detection showed an ME lower than 5% and resulted very sensitive (MQL equal to 0.5 µg/kg for At and Sc) and accurate (Table S2) [5]. In the study of Romera-Torres et al. [32], magnesium sulphate combined with GBC was selected for the clean-up step, obtaining a clearer and colorless extract, but the negative matrix effect was not avoided (ME lower than −50%).

On the other hand, in milk, eggs and porcine muscle samples, Zheng et al. [30] tested different solvents to improve extraction efficiency and purification protocols, which is of great importance in these kinds of samples due to their high content of fat, proteins and endogenous substances. Acetonitrile and acetonitrile mixtures with acetic, formic and trifluoroacetic acids were tested for their deproteinization capacity. The highest recoveries were achieved with a mixture of acetonitrile with a 0.5% of trifluoroacetic acid. In the clean-up step, C18 sorbent was useful for the removal of fats from the matrix, being the CEN QuEChERS purification methodology the most suitable for animal matrices. Recoveries between 74% and 99% were obtained with good RSD (≤7.7% for interday precision) indicating that the proposed method was accurate and precise (Table S2).

### 4.3. Application of New Materials in Sample Preparation

**Figure 5:**
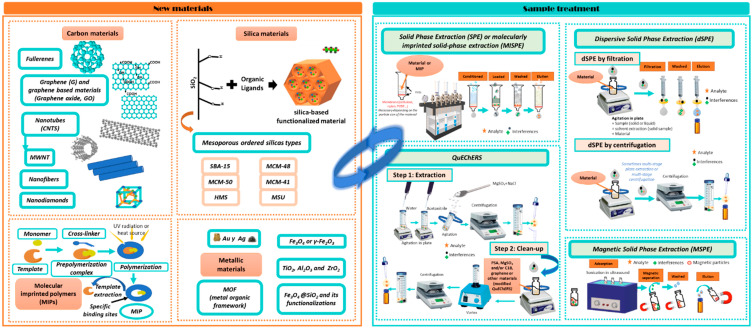
New materials used in sample preparation techniques.

Currently, the application of new materials in the food sample treatment for the analysis of organic compounds is having a great impact [47]. This is due to the new materials having advanced textural properties, including high surface area, large pore volume, controllable particle size and morphology, well-defined pore-size distribution, controllable wall composition and functionalization and excellent chemical, thermal and mechanical stability, among others, making them suitable sorbents for sample preparation. The most common materials are polymer-based materials such as the MIPs, OMSs; carbon-based materials such as graphene (G), graphene oxide (GO) and carbon nanotubes (CNTs); or metallic-based materials such as magnetic nanoparticles (MNPs) and metal-organic frameworks (MOFs). All these materials can be applied as sorbents in different sample treatment protocols such as SPE, dispersive SPE (dSPE), QuEChERS, molecularly imprinted SPE (MISPE), magnetic SPE (MSPE), etc. (Figure 5).

Different MIPs have been used for sample preparation in TAs analysis. These synthetic porous materials have specific recognition sites for analogous molecules (Figure 5). MIPs are obtained by polymerizing in bulk or by precipitation using a template and monomer, together with a crosslinking agent, an initiator of polymerization and a porogenic solvent, which leads to obtaining a highly crosslinked polymer [48]. When the template is removed, the cavity generated in the MIPs is specific to the analyte or other molecules with a similar structure. This is because the template is carefully selected, according to the analyte or analytes to be determined. In this sense, Zeng et al. [49] prepared an MIP by precipitation polymerization for the selective extraction and simultaneous determination of four TAs (At, Sc, anisodine and anisodamine). For this task, anidosine was used as template molecule, methacrylic acid (MAA) as functional monomer, trimethylolpropane trimethacrylate (TRIM) as cross-linker and acetonitrile as a porogen solvent. The MISPE process (with 100 mg of MIP) was applied to extract TAs from *Przewalskia tangutica* fruits, obtaining recoveries from 82 to 102% [49] (Table 7). In a more recent work, an MIP prepared by precipitation polymerization [50] but using Sc as a template, monoethyl fumarate (MFMA) as a functional monomer, ethylene dimethacrylate (EGDMA) as a cross-linker and 2,2-azobisisobutylnitrle (AIBN) as an initiator was also evaluated. MFMA was selected because it contributed to the enhancement of the adsorption capacity and selectivity of MIP, owing to the unique monomer structure [50,51]. The material obtained was applied in the MISPE procedure to determine Sc in plant samples obtaining recoveries between 96 and106% [50] (Table 7). The good recovery of Sc was maintained after 25 cycles, which indicates the good stability of the material.

Recently, OMSs are being highly used in different compounds such as xenobiotics [52], process contaminants [53] and natural toxins [14,54], and they can also be applied in different sorbent-based techniques such as SPE, dSPE or MSPE, among others [55]. In this sense, SBA-15 has been the main hexagonal mesostructured silica employed as sorbent for sample preparation. For example, in a recent paper, sulfonic-acid-functionalized SBA-15 (SBA-15-SO-_3_^−^ (Table 7) has been satisfactorily used as SCX phase in SPE (recoveries between 93 and105%) for the analysis of TAs in pseudo-cereals, cereals and legumes [14]. Almost no matrix effect was found for TAs in samples of corn, sorghum and teff flours. In addition, the SBA-15-SO_3_^−^ material showed better recoveries compared to a commercial SCX material, so it was a promising alternative to conventional clean-up sorbents. In other work [28], an HMS type mesostructured silica was synthesized and functionalized with sulfonic acid groups (HMS-SO_3_^−^) and compared with SBA-15-SO_3_^−^. Both materials were evaluated as SCX sorbents for sample extract clean-up by SPE and dSPE to determine At and Sc in commercial culinary aromatic herbs (unpublished results). Under optimized conditions, 0.25 g of sample were subject to SLE with acidified water (pH 1.0), and good recovery percentages were achieved for At and Sc using 75 mg of HMS-SO_3_^−^ in SPE as the clean-up stage prior to their determination by HPLC-MS/MS. The proposed method was validated, showing recoveries in the range of 70–92% and applied for the analysis of At and Sc of 16 commercial samples of thyme, basil and coriander.

## 5. Future Projections and New Directions

Compared to other contaminants, the use of new materials in food sample preparation for the analysis of natural toxins such as alkaloids is very scarce [56]. However, current trends in sample preparation involve moving towards “greener” approaches by scaling down analytical operations and integrating new advanced materials as sorbents. By scaling down the procedures, it is possible to develop less time-consuming and more cost-effective analytical methods to extract natural toxins from food samples. In this regard, some analytical strategies based on the use of reduced amount of new materials and/or microextraction techniques have been developed [55]. For example, in the recent work of our group, the miniaturization and modification of the QuEChERS protocol using different OMSs as dispersive clean-up sorbents for the extraction of 21 pyrrolizidine alkaloids from aromatic herbs was demonstrated [57]. The procedure was miniaturized by reducing the amounts of sample (0.2 g), solvents (2 mL), clean-up sorbents (25 mg sorbent + 150 mg MgSO4) and partitioning salts (0.65 g) employed. The best results achieved OMSs functionalized with -NH_2_ groups compared to conventional PSA as a sorbent. The method was validated showing very good recoveries (73–105%).

For TAs analysis, a crystalline porous material (MOF) prepared by binding metal ions with organic binding ligands through coordination reactions was evaluated in Chinese herbal tablets [58]. The new material combined with a tropine-based ionic liquid (IL@MOF) was used as a sorbent for the preconcentration of At, Sc and anisodamine. Using 5 mg of the hybrid IL/MOD composite by dSPE (1 min of agitation), the recoveries obtained ranged from 92 to 105% [58]. In addition, the sorbent material has great potential for reusability and was demonstrated to possess excellent stability. The findings from the study of Yohannes at al. [58] provide insights into the preparation of novel sorbent materials that could be used for the development of food sample preparation protocols.

## 6. Conclusions

From 2015 to today, many efforts have been made to address the food safety issue of TAs. In this sense, due to the food alerts in recent years, maximum concentration levels have been regulated for food products likely to be contaminated with TAs, such as processed cereal-based foods and baby foods for infants and young children, unprocessed or processed millet, sorghum, maize, maize for popping, buckwheat and herbal infusions. In this sense, cereals and related products has been the food category most extensively analyzed within the last years, whereas the highest TAs levels have been found in teas and herbal teas. However, it is also necessary to determine the occurrence of these compounds in other food matrices less studied to date or which have not been considered in the new Commission Regulation (EU) 2021/1408 such as legumes, aromatic herbs, spices and vegetables. In addition, there is lack of information relating to the effect of food processing and culinary preparation on the presence of TAs in order to achieve a reliable assessment of the real intake of these alkaloids by the population and improve the risk management of these contaminants. On the other hand, solid–liquid extraction is still the technique most widely used for sample preparation, although others based on the use of sorbents are also being applied. In addition, aiming to improve the extraction performance of sample preparation techniques, some new sorbents such as molecularly imprinted polymers, mesostructured silica-based materials and metal–organic frameworks have been prepared and evaluated for TAs analysis. This opens up a huge research window for the years ahead to develop analytical methods for the analysis of these toxins.

## Figures and Tables

**Table 2:** Occurrence of TAs in teas, herbal teas, herbal supplements, aromatic herbs and spices.

Foods (Nº of Samples Analyzed)	Nº of Samples with At (Range)	Nº of Samples with Sc (Range)	Other TAs	[Ref.]
Green tea, black tea, chamomile, fennel, melissa, peppermint and rooibos (70 samples)	1 of fennel (83 µg/kg) 8 of peppermint (20–208 µg/kg)	1 of fennel (11 µg/kg) 8 of peppermint (20–208 µg/kg) 1 of chamomile (2.1 µg/kg) 1 of rooibos (2 µg/kg)	N.St	[27]
Dry (herbal) teas (121 samples)	85 of dry (herbal) teas (0.0067–294.8 µg/kg)	85 of dry (herbal) teas (0.0067–133.7 µg/kg)	6-HO-tropinone, Nortropinone, Pseudotropine, Scopine, Scopoline, Tropine, tropinone, convolamine, convolidine, convolvine, fillalbine, anisodamine, apoatropine, aposcopolamine, homatropine, littorine, noratropine, norscopolamine	[21]
Green tea with mint, green tea with mate, Piccadilly tea, red Chinese tea, rooibos of apple, relaxing tea, *Mentha pulegium* tea, ayurvedic spiced tea, coca leaf tea, mixture of teas (11 samples)	1 of *Mentha pulegium* tea (9 µg/kg) 1 of coca leaf tea (27 µg/kg)	N.D	Physoperuvine, pseudotropine, tropine, homatropine, apoatropine	[26]
Herbal teas, herbal supplements (60 samples)	1 of herbal tea with *Melissa officinalis* and others (25 µg/kg) 1 of herbal tea with *Taraxacum officinalis* and others (69 µg/kg)	1 of herbal tea with *Melissa officinalis* and others (50 µg/kg)	Homatropine, anidosamine	[25]
Thyme, basil and coriander (16 samples)	5 of thyme (<5–5.7 µg/kg) 5 of basil (9–11.7 µg/kg) 4 of coriander (9.9–42 µg/kg)	2 of thyme (<5 µg/kg) 1 of coriander (34 µg/kg)	N.St	[28]

Abbreviations: At: atropine. N.D: not detected or below the limit of detection. N.St: not studied. Sc: scopolamine. TAs: tropane alkaloids.

**Table 3:** Occurrence of TAs in animal-derived foods.

Foods (Nº of Samples Analyzed)	Nº of Samples with At (Range)	Nº of Samples with Sc (Range)	Other TAs	[Ref.]
Honey (40 samples)	9 of honey (1.4–3.8 µg/kg)	N.D	N. St	[5]
Porcine muscle, egg, milk (30 samples)	N.D	N.D	N.St	[30]
Honey (23 samples)	2 of honey (0.012 µg/kg)	1 of honey (0.012 µg/kg)	N.St	[33]
Honey (19 samples)	N.D	2 of honey (27 µg/kg)	Physoperuvine, tropine, pseudotropine, tropinone	[32]

Abbreviations: At: atropine. N.D: not detected or below the detection limit. N. St: not studied. Sc: scopolamine. TAs: tropane alkaloids.

**Table 4:** Summary of methodologies that applied solid–liquid and liquid–liquid extraction protocols for the analysis of TAs in foods.

Analyte	Sample (Amount)	Extraction Conditions	Other Treatments	Recovery (%)	[Ref.]
At, Sc + 4 TAs + others (ergot alkaloids)	Breakfast cereals, breakfast cereals with milk, biscuits, cookies (4 g)	40 mL MeOH/ H_2_O /FA (60:40:0.4, *v*/*v*/*v*) + agitation (30 min) + centrifuged (15 min, 3500 g)	Ultrafiltrated	86–91 TAs 88 At 88 Sc	[17]
At, Sc + 4 TAs + others (pyrrolizidine alkaloids)	Green tea, black tea, chamomile, fennel, melissa, peppermint and rooibos (1 g)	10 mL of aqueous HAC/MeOH solution (1:2, *v*/*v*) + agitation (30 min) + centrifuged (10 min, 4000 g)	Diluted with NH_4_OH + evaporated with N_2_ at 40 °C + reconstituted in H_2_O + filtered	80–95 TAs	[27]
At, Sc	Buckwheat flour, pasta and bakery (5 g)	25 mL MeOH/H_2_O (3:2, *v*/*v*) with 0.2% FA + 0.2% ACN + agitation (90 min) + centrifuged (15 min, 4000 rpm)	Diluted with the extraction solvent	88–103 At 83–103 Sc	[13]
At, Sc + 12 TAs + others (calystegines)	Bread (3 g)	30 mL H_2_O (HAC 0.5%)/MeOH (1:2, *v*/*v*) + vortex (1 min) + agitation (30 min) + centrifuged (10 min, 5000 rpm)	-	75–101 TAs 93 At 88 Sc	[39]
At, Sc + 11 TAs + others (cocaine, calystegines)	Pasta (3 g)	30 mL MeOH/H_2_O (2:1, *v*/*v*) with 0.5% HAC + Polytron (30 s) + agitation (30 min) + centrifuged (10 min, 4480 rcf)	Filtered	74–98 TAs 93 At 93 Sc	[40]
At, Sc + 2 TAs	Herbal teas, herbal supplements (1 g)	25 mL ACN/H_2_O (3:2, *v*/*v*) with 0.2% FA + agitation (90 min) + centrifuged (15 min, 2150 g × 2)	Diluted with the extraction solvent	83–107 TAs 105–107 At 83–103 Sc	[25]
At, Sc + others (pesticides, mycotoxins, growth regulators, pyrrolizidine alkaloids)	Oats and wheat (5 g)	10 mL ACN/ H_2_O (80:20, *v*/*v*) + shaking (2 min) + agitation (30 min) + centrifuged (5 min, 3000 rpm)	Filtered	75–119 At 72–116 Sc	[41]
At, Sc + 4 TAs + others (ergot alkaloids)	Bread (wheat, multi-grain, rye, wheat-rye) (4 g)	40 mL MeOH/H_2_O/FA (60:40:0.4, *v*/*v*/*v*) + agitation (30 min) + centrifuged (15 min, 3500 g)	Ultrafiltrated	73–94 TAs 83 At 73 Sc	[23]
At, Sc	Honey (5 g)	10 mL sodium acetate solution + vortex (2500 rpm, 15 min) + 10 mL ACN + centrifugation (10 min, 3000 rpm)	-	87–103 At 87–106 Sc	[33]
At, Sc + 19 TAs + others (pyrrolizidine alkaloids)	Sorghum, oregano, herbal tea (1 g)	10 mL MeOH/H_2_O/FA (60:39.6:0.4, *v*/*v*/*v*) + agitation (30 min) + centrifuged (5 min, g)	Filtered	78–115 TAs 95–111 At 84–110 Sc	[38]
At, Sc + others (mycotoxins, plant growth regulators, pesticides)	Wheat, barley, rice, oats, spelt, rye (2.5 g)	10 mL ACN/H_2_O/FA (79:20:1, *v*/*v*/*v*) + agitation (30 min) + centrifuged (3 min, 1902 g)	Filtered and diluted with ACN	103 At 105 Sc	[42]

Abbreviations: ACN: acetonitrile. At: atropine. FA: formic acid. HAC: acetic acid. MeOH: methanol. Sc: scopolamine. TAs: tropane alkaloids. -: Other treatmeants have not been applied.

**Table 5:** Summary of methodologies that applied solid-phase extraction protocols for the analysis of TAs in foods.

Analyte	Sample (Amount)	SLE Conditions	SPE Conditions	Other Treatments	Recovery (%)	[Ref.]
At, Sc + 22 TAs	Flours (buckwheat, millet and corn), cereal-based food (breakfast cereals, biscuits, pastry, pasta and bread), legumes, stir-fry mixes, dry herbal teas (4 g)	40 mL MeOH/H_2_O/FA (75:25:0.4, *v*/*v*/*v*) + agitation (30 min) + centrifuged (15 min, 3500 rpm)	OASIS MCX (150 mg) or Strata-X (200 mg) C: MeOH (6 mL) + MeOH/H_2_O/FA (75:25:1, *v*/*v*/*v*) (6 mL) L: supernatant (10 mL, in teas 5 mL) W: MeOH/H_2_O/ FA (75:25:1, *v*/*v*/*v*) (6 mL) Dry under vacuum (5–10 min) E: MeOH with 0.5% NH_4_OH (aq)	Evaporated with N_2_ in a warmed water bath + reconstituted in MeOH/H_2_O (10:90, *v*/*v*) + filtered	20–124 TAs 64–112 At 51–117 Sc	[21]
At, Sc + 22 TAs	Herbal tea infusions (37.5 mL)	-	OASIS MCX (150 mg) C: MeOH (6 mL) + H_2_O with 1% FA (6 mL) L: 37.5 mL infusion (+ 75 µL FA + centrifuged) W: MeOH/H_2_O/ FA (75:25:1, *v*/*v*/*v*) (6 mL) Dry under vacuum (5–10 min) E: MeOH with 0.5% NH_4_OH (aq)	Evaporated with N_2_ in a warmed water bath + reconstituted in MeOH/H_2_O (10:90, *v*/*v*) + filtered	51–104 TAs 92 At 90 Sc	[21]
At, Sc + 11 TAs	Buckwheat, buckwheat flour and pasta; soy and soy flour; peeled millet and millet flour; linseed and linseed flour (1 g)	10 mL H_2_O (HAC 0.5%)/MeOH (1:2, *v*/*v*) + vortex (1 min) + agitation (30 min) + centrifuged (10 min, 5000 rpm)	Strata-X (200 mg) C: MeOH/ H_2_O (1% HAC) (2:1, *v*/*v*) L: supernatant (10 mL) W: MeOH/H_2_O (1% HAC) (2:1, *v*/*v*) E: MeOH with 3% NH_4_OH (aq)	Evaporated with N_2_ + reconstituted in MeOH/H_2_O (0.1% HAC) (50:50, *v*/*v*) + filtered	60–109 TAs 63–93 At 63–94 Sc	[16]
At, Sc + 11 TAs	Teas and herbal teas (1 g)	10 mL MeOH/H_2_O/FA (75:25:0.4, *v*/*v*/*v*) + agitation (30 min) + centrifuged (5 min, 5000 rpm)	Strata-X-C (200 mg) C: MeOH/ H_2_O/FA (75:25:1, *v*/*v*/*v*) L: supernatant (5 mL) W: MeOH/ H_2_O/FA (75:25:1, *v*/*v*/*v*) Dry under vacuum (1 h) E: MeOH with 3% NH_4_OH (aq)	Evaporated with N_2_ + reconstituted in H_2_O/MeOH (90:10 *v*/*v*) + filtered	75–128 TAs 99–113 At 96–122 Sc	[26]
At, Sc + 11 TAs	Cereal based baby foods (pap, biscuits, snacks and grissines) (1 g)	10 mL MeOH/H_2_O (2:1, *v*/*v*) with 0.5% HAC + vortex (1 min) + agitation (30 min) + centrifuged (10 min, 4480 rcf) and diluted tenfold with MeOH/H_2_O (2:1, *v*/*v*) with 1% HAC	Strata-X-C (20 × 2 mm, 25 µm) SPE online, 8 mL injected	-	66–98 TAs 68–95 At 83–93 Sc	[18]
At, Sc + 11 TAs + others (cocaine, calystegines)	Tea (3 g)	-	Strata-X-C (200 mg) C: MeOH (6 mL) and MeOH/H_2_O (1% HAC) (2:1, *v*/*v*) (6 mL) L: 45 mL infusion (+ cooled + centrifuged + acidified 1% HAC) W: MeOH/H_2_O (1% HAC) (2:1, *v*/*v*) (6 mL) E: MeOH with 3% NH_4_OH (aq) (6 mL)	-	74–98 TAs 93 At 93 Sc	[40]

Abbreviations: ACN: acetonitrile. At: atropine. C: conditioning. E: elution. FA: formic acid. HAC: acetic acid. L: loading. MeOH: methanol. N.S.: Not shown. SLE: solid-liquid extraction. SPE: solid phase extraction. Sc: scopolamine. TAs: tropane alkaloids. W: washing. -: SLE has not been used.

**Table 6:** Summary of methodologies that applied QuEChERS protocols for the analysis of TAs in foods.

Analyte	Sample (Amount)	Extraction Conditions	Clean-Up Conditions	Other Treatments	Recovery (%)	[Ref.]
At, Sc + others (pyrrolizidine alkaloids)	Honey (1.5 g)	10 mL H_2_SO_4_ (0.1 M) + 0.5 g of zinc dust + agitation (1 h 30 min) + centrifuged (10 min, 3700 g) = supernatant + 10 mL ACN + Q-Sep QuEChERS extraction salts: MgSO_4_ (4 g), C_6_H_5_Na_3_O_7_ 2H_2_O (1 g), C_6_H_6_Na_2_O_7_ 1.5 H_2_O (0.5 g), NaCl (1 g) + shaken (4 min) + centrifuged	PSA (0.15 mg), MgSO_4_ (0.9 g). Vortex + centrifuged	Evaporated under vacuum at 45 °C + reconstituted in ACN/H_2_O (0.1% FA) (13:87, *v*/*v*)	101–104 At 96–109 Sc	[5]
At, Sc	Buckwheat, buckwheat flour and pasta, soy, wheat, amaranthus grain, chia seeds, peeled millet (5 g)	10 mL H_2_O + vortex (1 min) + 10 mL of ACN with 1% FA + vortex (2 min) + Na_2_SO_4_ (4 g) + NH₄CH₃CO₂ (1 g) + centrifuged (5 min, 5000 rpm)	PSA (25 mg), GBC (25 mg). Vortex (1 min) + centrifuged (5 min, 5000 rpm)	Diluted with H_2_O + filtered	66–92 At 50–88 Sc	[22]
Sc, L-hyoscyamine + other (sparteine)	Porcine muscle (2 g) Egg and milk (2 mL)	0.1 mL EDTA + 10 mL of ACN with 0.5% TFA + vortex 5 min + MgSO_4_ (4 g) + NaCl (1 g) + C_6_H_5_Na_3_O_7_ 2H_2_O (1 g) + C_6_H_6_Na_2_O_7_·1.5 H_2_O (0.5 g) + vortex (5 min) + centrifuged (10 min, 2600 g)	C18 (0.15 g), MgSO_4_ (0.9 g) Vortex (5 min) + centrifuged (10 min, 2600 g)	Evaporated with N_2_ at 45 °C + reconstituted in MeOH + vortex + centrifuged + filtered	83–99 At 74–99 Sc	[30]
At, Sc + 9 TAs	Honey (2.5 g)	10 mL MeOH/ H_2_O/FA (75:25:0.4, *v*/*v*/*v*) + agitation (30 min) + centrifuged (10 min, 5000 rpm)	MgSO_4_ (0.3 g), GBC (0.05 g). Vortex (1 min) + centrifuged (10 min, 5000 rpm)	Filtered	71–120 TAs 85–103 At 116–120 Sc	[32]
At, Sc	Wheat, corn, rice, oat and millet flours, mixed cereals flours, infant cereals, cereal-based products (5 g)	10 mL H_2_O + shaken + 10 mL of ACN with HAC (95:5, *v*/*v*) + agitation (30 min) + QuEChERS salt mixture: NaCl (1 g) + MgSO_4_ (4 g) + shaken + centrifuged (10 min, 3500 rpm) = 5 mL + hexane + shaken + centrifuged (1 min, 4000 g)	The hexane phase was discarded	Evaporated with N_2_ at 40 °C + reconstituted in MeOH + sonicated + diluted with H_2_O + vortex + centrifuged	95–107 At 87–118 Sc	[19]
At, Sc	Spinach-based products (10 g)	10 mL of ACN + NaCl (1 g) + MgSO4 (4 g) + C_6_H_5_Na_3_O_7_ 2H_2_O (1 g) + C_6_H_6_Na_2_O_7_ 1.5 H_2_O (0.5 g) + shaken (1 min) + centrifuged (5 min, 3500 rpm) = 5 mL	PSA (0.25 mg), MgSO_4_ (0.75 g). Shaken (30 s) + centrifuged (5 min, 3500 rpm)	Diluted with H_2_O + Filtered	94–103 At 91–98 Sc	[29]

Abbreviations: ACN: acetonitrile. At: atropine. DSPE: dispersive solid phase extraction. EDTA: ethylenediaminetetraacetic acid. FA: formic acid. GBC: graphitized black carbon. HAC: acetic acid. MeOH: methanol. PSA: primary secondary amine. Sc: scopolamine. TAs: tropane alkaloids. TFA: trifluoroacetic acid.

**Table 7:** Summary of methodologies that used new materials in the sample preparation for the analysis of TAs.

Analyte	Sample (Amount)	SLE Conditions	SPE Conditions	Other Treatments	Recovery (%)	[Ref.]
At, Sc + 2 TAs	*Przewalskia tangutica* fruit (1 g)	3 mL NH_3_ + vortex (2 min) + 30 mL ACN + agitation (30 min) + centrifuged (5 min, 8000 rpm) × 3 Evaporated + dissolved in MeOH (1 mL) before MISPE	MIP (100 mg) C: ACN (3 mL) L: supernatant (1 mL) W: ACN saturated n-hexane (1 mL) E: HAC/MeOH (3/7, *v*/*v*) (2 mL)	-	82–102 TAs 97–102 At 95–98 Sc	[49]
Sc	Plants (Hindu *Datura*, Belladona and *Hyoscyamus niger*) (50 mg)	250 mL HCl (2 M) + ultrasound (2 h) 10 mL supernatant + NH_3_ (pH 8–9) + dichloromethane + dried + dissolved with MeOH (50 mL) before MISPE	MIP (100 mg) C: MeOH (10 mL) + dichloromethane (5 mL) L: supernatant (10 mL) W: MeOH/ethyl acetate (10/90, *v*/*v*) (5 mL) E: MeOH/H_2_O (2% HAC) (60/40, *v*/*v*) (2 mL)	Evaporated + reconstituted in dichloromethane	96–106	[50]
At, Sc	Gluten-Free grains and flours (1 g)	8 mL H_2_O (1.1% HCl, pH 1.0) + agitation (30 min) + centrifuged (10 min, 6000 rpm) + precipitate washing 1 mL H_2_O (1.1% HCl, pH 1.0) + filtration before SPE	M-SBA-15-SO_3_^−^ (150 mg) C: H_2_O (1.1% HCl, pH 1.0) (5 mL) L: supernatant (9 mL) W: H_2_O (1.1% HCl, pH 1.0) (3 mL) E: MeOH (3 mL) + MeOH with 10% ammonia solution (90:10, *v*/*v*, pH 11.8) (6 mL)	Evaporated under vacuum + reconstituted in ACN/H_2_O (50:50, *v*/*v*)	93–105 At 93–96 Sc	[14]
At, Sc	Thyme, basil, coriander (0.25 g)	8 mL H_2_O (1.1% HCl, pH 1.0) + agitation (30 min) + centrifuged (10 min, 6000 rpm) + precipitate washing 1 mL H_2_O (1.1% HCl, pH 1.0) + filtration before SPE	HMS-SO_3_^−^ (75 mg) C: H_2_O (1.1% HCl, pH 1.0) (5 mL) L: supernatant (9 mL) W: H_2_O (1.1% HCl, pH 1.0) (3 mL) E: MeOH (3 mL) + MeOH with 10% ammonia solution (90:10, *v*/*v*, pH 11.8) (6 mL)	Evaporated under vacuum + reconstituted in ACN/H_2_O (50:50, *v*/*v*)	87–92 At 70–92 Sc	[28]

Abbreviations: ACN: acetonitrile. At: Atropine. C: conditioning. E: elution. HAC: acetic acid. L: Loading. MeOH: Methanol. MIP: molecularly imprinted polymer. MISPE: molecularly imprinted solid-phase extraction. Sc: scopolamine. SLE: solid-liquid extraction. SPE: solid phase extraction. TAs: tropane alkaloids. W: washing. -: Other treatmeants have not been applied.

## Supplementary Materials

The following supporting information can be downloaded at: https://www.mdpi.com/article/10.3390/foods11030407/s1, Table S1: RASFF notifications from 2015 to 2021 on atropine and scopolamine in food samples. Table S2: Chromatographic conditions and validation parameters for the analysis of TAs. Figure S1: RASFF notifications of TAs by food category and food type (from 2015 to 2021).
