# Supplementary material for: Occurrence and Chemistry of Tropane Alkaloids in Foods, with a Focus on Sample Analysis Methods: A Review on Recent Trends and Technological Advances
Source: Foods. 2022 Jan 30;11(3):407. doi: 10.3390/foods11030407 (PMC8833975; doi:10.3390/foods11030407)
Supplement: Supplementary file 1 [file foods-11-00407-s001.zip › foods-1552881-supplementary.pdf]

SUPPLEMENTARY MATERIALS

Occurrence and Chemistry of Tropane Alkaloids in Foods, with a Focus on Sample Analysis Methods: A Review on Recent Trends and Technological Advances

Table S1. RASFF notifications from 2015 to 2021 on atropine and scopolamine in food samples.

Date	Notification country	Product Category	Subject	Action taken	Distribution status	Risk Decision
20/02/2015	Austria	Cereals and bakery products	Atropine (0.062 mg/kg - ppm) and scopolamine (0.033 mg/kg - ppm) in brown millet from Austria	Withdrawal from the market	Distribution to other member countries	Serious
20/02/2015	Germany	Cereals and bakery products	Atropine (156.2; 207.5 µg/kg - ppb) and scopolamine (27.2; 31.3 µg/kg - ppb) in organic polenta cornmeal from Germany	Withdrawal from the market	Distribution to other member countries	Serious
20/03/2015	Austria	Cereals and bakery products	Atropine (0.481 mg/kg - ppm) and scopolamine (0.533 mg/kg - ppm) in organic millet dumplings from Hungary	Withdrawal from the market	Distribution restricted to notifying country	Serious
20/03/2015	Austria	Cereals and bakery products	Atropine (0.304 mg/kg - ppm) and scopolamine (0.358 mg/kg - ppm) in millet dumplings from Hungary	Withdrawal from the market	Distribution restricted to notifying country	Serious
30/03/2015	Austria	Cereals and bakery products	Atropine (26 µg/kg - ppb) and scopolamine (11 µg/kg - ppb) in millet honey poppies from Germany	Withdrawal from the market	No distribution from notifying country	Serious
30/03/2015	Austria	Cereals and bakery products	Atropine (30 µg/kg - ppb) and scopolamine (24 µg/kg - ppb) in gluten-free millet from Austria	Withdrawal from the market	Distribution to other member countries	Serious

31/03/2015	Germany	Cereals and bakery products	Atropine (384 μg/kg - ppb) and scopolamine (388 μg/kg - ppb) in millet balls from Hungary	Recall from consumers	Distribution to other member countries	Serious
04/06/2015	Germany	Cereals and bakery products	Atropine (198.5 μg/kg - ppb) and scopolamine (45 μg/kg - ppb) in organic polenta cornmeal from Germany	Recall from consumers	Distribution to other member countries	Serious
18/09/2015	Germany	Cereals and bakery products	Atropine (29 μg/kg - ppb) and scopolamine (6 μg/kg - ppb) in microwave popcorn from Spain	Recall from consumers	Distribution to other member countries	Serious
26/11/2015	Czech Republic	Cereals and bakery products	Atropine (1200; 1500 μg/kg - ppb) and scopolamine (360; 460 μg/kg - ppb) in sorghum flour from the Czech Republic with raw material from the Czech Republic and Slovakia	Withdrawal from the market	Distribution to other member countries	Serious
01/02/2016	Czech Republic	Cereals and bakery products	Atropine (180; 130 μg/kg - ppb) and scopolamine (36;27 μg/kg - ppb) in gluten-free baking mix and related products based on sorghum from the Czech Republic	Destruction	Distribution to other member countries	Serious
13/04/2016	Czech Republic	Dietetic foods, food supplements, fortified foods	Atropine (7.9 μg/kg - ppb) in baby porridge without milk from Spain	Withdrawal from the market	Distribution to other member countries	Undecided
22/07/2016	Austria	Cereals and bakery products	Atropine (10.5; 12.2; 12.3 μg/kg - ppb) and scopolamine (2.0; 3.8; 3.1 μg/kg - ppb) in corn from Germany	Withdrawal from the market	Distribution to other member countries	Serious
20/09/2016	Austria	Cereals and bakery products	Atropine (23.5 μg/kg - ppb) and scopolamine (9.5 μg/kg - ppb) in millet flour from Hungary	Public warning - press release	Distribution to other member countries	Serious
28/12/2016	Czech Republic	Cocoa and cocoa preparations, coffee and tea	Atropine (206.4 μg/kg - ppb) and scopolamine (31.7 μg/kg - ppb) in herbal tea from Poland	Withdrawal from the market	Distribution to other member countries	Serious
02/06/2017	Germany	Cocoa and cocoa preparations, coffee and tea	Atropine (0.052 %) in organic burdock root (*Arctium lappa*) tea from Germany, processed in Croatia, with raw material from Albania	Withdrawal from the market	Distribution restricted to notifying country	Serious
24/02/2017	Czech Republic	Cocoa and cocoa preparations, coffee and tea	Atropine (72 μg/kg - ppb) and scopolamine (23 μg/kg - ppb) in herbal tea with Echinacea from Poland	Return to consignor	Distribution to other member countries	Serious

07/06/2017	United Kingdom	Dietetic foods, food supplements, fortified foods	Atropine and scopolamine in dried herbs (*Ruscus aculeatus*) from Bulgaria	No action taken	Distribution to other member countries	Serious
21/03/2018	Hungary	Nuts, nut products and seeds	Atropine (16177.6 µg/kg - ppb) and scopolamine (4658.3 µg/kg - ppb) in whole cumin seeds from Hungary	Withdrawal from the market	Distribution to non-member countries	Serious
24/05/2018	Germany	Cereals and bakery products	Atropine (6.60 µg/kg - ppb) and scopolamine (1.77 µg/kg - ppb) in popcorn from France	Withdrawal from the market	Distribution to other member countries	Serious
05/06/2018	Germany	Cereals and bakery products	scopolamine (10.3 µg/kg - ppb) in popcorn in grain from Argentina	Recall from consumers	distribution to other member countries	Serious
16/07/2018	Czech Republic	Herbs and spices	Atropine (213 µg/kg - ppb) and scopolamine (44.7 µg/kg - ppb) in herbal infusion from Poland	Withdrawal from the market	Distribution to other member countries	Serious
26/09/2018	Germany	Cereals and bakery products	Atropine (60.7 µg/kg - ppb) and scopolamine (38.8 µg/kg - ppb) in organic muesli from Austria	Recall from consumers	Distribution to other member countries	Serious
20/12/2018	France	Cereals and bakery products	Atropine (53 µg/kg - ppb) and scopolamine (20 µg/kg - ppb) in organic buckwheat flour from France		Distribution to other member countries	Serious
29/01/2019	Croatia	Cocoa and cocoa preparations, coffee and tea	Atropine (200.5 µg/kg - ppb) and scopolamine (488.7 µg/kg - ppb) in peppermint from Serbia	Return to consignor	Product (presumably) no longer on the market	Serious
01/04/2019	Croatia	Cereals and bakery products	Atropine (4.5 µg/kg - ppb) and scopolamine (4.3 µg/kg - ppb) in corn grits from Serbia	Public warning - press release	Distribution restricted to notifying country	Serious
06/08/2019	Netherlands	Cereals and bakery products	Atropine (2.2 µg/kg - ppb) and scopolamine (2.7 µg/kg - ppb) in breakfast porridge 8 cereals with honey from Spain	Withdrawal from the market	Distribution restricted to notifying country	Undecided
26/08/2019	France	Cereals and bakery products	Atropine (47 µg/kg - ppb) and scopolamine (30 µg/kg - ppb) in organic buckwheat flour from France	Recall from consumers	Distribution to other member countries	Serious

24/01/2020	Germany	Cereals and bakery products	Atropine (19 µg/kg - ppb) and scopolamine (6.4 µg/kg - ppb) in organic soy flakes from Austria, with raw material from the Czech Republic	Withdrawal from recipient (s)	Distribution to other member countries	Serious
25/05/2020	Germany	Cocoa and cocoa preparations, coffee and tea	Atropine (543.1 µg/kg - ppb) and scopolamine (31.4 µg/kg - ppb) in organic blackberry leaves from Bulgaria	Withdrawal from the market	Product (presumably) no longer on the market	Serious
04/09/2020	Germany	Cereals and bakery products	Atropine (24.2 µg/kg - ppb) and scopolamine (13.3 µg/kg - ppb) in wild brown millet from Germany	Withdrawal from recipient (s)	distribution to non-member countries	Undecided
04/11/2020	Germany	Herbs and spices	Atropine (26.8 µg/kg - ppb) and scopolamine (39.9 µg/kg - ppb) in peppermint from Turkey	Recall from consumers	Distribution restricted to notifying country	Not serious
11/12/2020	Germany	Cereals and bakery products	Atropine (36,0 ± 16,28 µg/kg - ppb) and scopolamine (22,4 ± 8,48 µg/kg - ppb) in ground wild brown millet from Germany	Withdrawal from the market	Distribution to other member countries	Undecided
18/01/2021	Germany	Cereals and bakery products	High levels of scopolamine (1.2; 1.9 µg/kg - ppb) and atropine (23; 35 µg/kg - ppb) in popcorn maize from Spain	Withdrawal from the market	No distribution from notifying country	Serious
17/03/2021	Slovakia	Fruits and vegetables	Foodborne outbreak (tropane alkaloids) caused by, atropine (1938 µg/kg - ppb) and scopolamine (hyoscine ((-)-scopolamine): 1164 µg/kg - ppb) in deep frozen spinach puree from Slovakia	Withdrawal from the market	Distribution to other member countries	Serious

Table S2. Chromatographic conditions and validation parameters for the analysis of TAs.

**Alkaloids** *Samples*	**Sample preparation**	**Analytical technique (analyser)**	**Stationary phase type** **Brand (*dimensions, particle size*)** **Mobile phase**	**Run time (min)**	**Linear range (µg/kg)**	**MDL (µg/kg)**	**MQL (µg/kg)**	**RSD (%)**	**Recovery (%)**	**[Ref.]**
At, Sc + 4 TAs + others (ergot alkaloids) *Breakfast cereals, breakfast cereals with milk, biscuits, cookies*	SLE	HPLC-MS/MS (QqQ)	C18 Waters Xbridge C18 (*150 x 3.1 mm ID, 5 µm*) A: H_2O, B: ACN:H_2O (90:10 *v/v*), both with 6.5 mM NH_4OH	17	0-50	0.2-0.5 TAs 0.3 At 0.3 Sc	N.S	4-11 TAs 6 At 8 Sc	86-91 TAs 88 At 88 Sc	[17]
At, Sc + 22 TAs *Flours (buckwheat, millet and corn), cere-al-based food (break-fast cereals, biscuits, pastry, pasta and bread), legumes, stir-fry mixes, dry herbal teas*	SLE-SPE	HPLC-MS/MS (QqQ)	C18 Waters UPLC BEH C18 (*150 x 2.1 mm ID, 1.7 µm*) A: H_2O with 10 mM $(NH_4)_2CO_3$ pH 10 with ammonia, B: ACN	15	0-50	0.05-1 TAs 0.05-0.2At 0.05-0.2 Sc	0.5-5 TAs 0.5-1 At 0.5-1 Sc	2-54 TAs 7-16 At 6-48 Sc	20-124 TAs 64-112 At 51-117 Sc	[21]
At, Sc + 22 Tas *Herbal tea infusions*	SLE-SPE	HPLC-MS/MS (QqQ)	C18 Waters UPLC BEH C18 (*150 x 2.1 mm ID, 1.7 µm*) A: H_2O with 10 mM $(NH_4)_2CO_3$ pH 10 with ammonia, B: ACN	15	0-50	0.0017-0.013* TAs 0.0017* At 0.0017* Sc	0.0067-0.033* TAs 0.0067* At 0.0017* Sc	2-44 TAs 6At 6 Sc	51-104 TAs 92 At 90 Sc	[21]
At, Sc + 4 TAs + others (pirrolizidine alkaloids) *Teas and Herbal teas Green tea, black tea, chamomile, fennel, melissa, peppermint and rooibos*	SLE	HPLC-MS/MS (QTRAP)	C18 Hypersil Gold C18 (*50 x 2.1 mm ID, 1.9 µm*) A: H_2O with 0.5% FA, B: MeOH/H_2O/FA (94.5/5/0.5 *v/v/v*), both with oxalic acid and 2 mM $NH_4CH_3CO_2$	10	1-500	N.S	1 At 1 Sc	N.S	80-95 TAs	[27]

At, Sc + 11 Tas<br>*Buckwheat, buck-wheat flour and pasta; soy and soy flour; peeled millet and mil-let flour; linseed and linseed flour*	SLE-SPE	HPLC-HRMS (Orbitrap)	HILIC coupled C18<br>Zorbax Eclipse Plus HILIC (100 x 2.1 mm ID, 3.5 µm)<br>Zorbax Eclipse Plus C18 (100 x 2.1 mm ID, 1.8 µm)<br>A: ACN, B: H_2O with 0.1 % FA	18	0.5-100	0.1-2 TAs<br>0.5 At<br>1 Sc	0.5-3 TAs<br>1 At<br>2 Sc	1-20<br>3-13 At<br>3-9 Sc	60-109 TAs<br>63-93 At<br>63-94 Sc	[16]
At, Sc<br>*Buckwheat, buck-wheat flour and pasta, soy, wheat, amaranthus grain, chia seeds, peeled millet*	Modified QuEChERS	UHPLC-MS/MS (QqQ)	C18<br>Zorbax Plus C18 (*100 x 2.1 mm ID, 1.8 µm*)<br>A: ACN, B: H_2O with 0.1 % FA	10	0.1-100	0.2 At<br>0.04 Sc	2 At<br>0.4 Sc	2-17 At<br>2-11 Sc	66-92 At<br>50-88 Sc	[22]
At, Sc + others (pirrolizidine alkaloids)<br>*Honey*	QuEChERS	HPLC-HRMS (Q-Orbitrap)	C8<br>*Ascentis Express C8 column (150 x 3 mm, ID, 2.7 µm)*<br>A: H_2O with 0.1 % FA<br>B: MeOH:ACN (1:1 *v/v*)	25	0-10.5*	0.1 At<br>0.2 Sc	0.5 At<br>0.5 Sc	1-4 At<br>3-6 Sc	101-104 At<br>96-109 Sc	[5]
At, Sc<br>*Buckwheat flour, pasta and bakery*	SLE	UHPLC-MS/MS (QqQ)	C18<br>C18 Kinetex<br>(*100 x 2.1 mm, ID, 2.6 µm*)<br>A: H_2O, B: MeOH both with 0.2 % FA	20	0.05-1	0.09 At<br>0.03 Sc	0.3 At<br>0.1 Sc	2-11 At<br>2-5 Sc	88-103 At<br>83-103 Sc	[13]
At, Sc + 11 Tas<br>*Teas and herbal teas*	SLE-SPE	HPLC-HRMS (Orbitrap)	HILIC coupled C18<br>Zorbax Eclipse Plus HILIC (*100 x 2.1 mm ID, 1.8 µm*) and Zorbax Eclipse Plus C18 (*100 x 2.1 mm ID, 1.8 µm*)<br>A: H_2O with 0.1% FA<br>B: ACN	18	5-100	N.S	5 At<br>10 Sc	1-26 TAs<br>1-9 At<br>4-25 Sc	75-128 TAs<br>99-113 At<br>96-122 Sc	[26]

At, Sc + 12 TAs + others (calystegines)<br>*Bread*	SLE	HPLC-HRMS (Orbitrap)	C18<br>Zorbax Eclipse Plus C18 (*100 x 2.1 mm ID, 1.8 μm*)<br>A: H_2O with 0.1% FA<br>B: MeOH	14	1-5000	N.S	N.S	5 At<br>4 Sc	75-101 TAs<br>93 At<br>88 Sc	[39]
At, Sc + 11 Tas<br>*Cereal based baby foods (pap, biscuits, snacks and grissines)*	SPE-Online	SPE-MS/MS (Orbitrap/QqQ)	C18<br>ACE Excel 3 Super C18 *(150 x 4.6 mm ID, 3 μm)*<br>MeOH with 0.75% NH_4OH	Orbi-trap 15.8<br>QqQ 17.3	0.5-50	0.5-5 TAs<br>0.5At<br>1-2.5 Sc	0.5-10 TAs<br>0.5-1 At<br>2.5-5 Sc	5 At<br>4 Sc	66-98 TAs<br>68-95 At<br>83-93 Sc	[18]
At, Sc + 11 TAs + others (cocaine, calystegines)<br>*Tea and pasta samples*	SLE Pasta<br>SLE-SPE Tea	HPLC-HRMS (Orbitrap)	C18<br>Zorbax Eclipse Plus C18 (*100 x 2.1 mm ID, 1.8 μm*)<br>A: H_2O with 0.1% FA, B: MeOH	14	1-5000	0.5-50 TAs<br>1 At<br>2 Sc	1-75 TAs<br>2.5 At<br>5 Sc	1-13 TAs<br>2 At<br>3 Sc	74-98 TAs<br>93 At<br>93 Sc	[40]
At, Sc + others (pesticides, mycotoxins, growth regulators, pyrrolizidine alkaloids)<br>*Oats and wheat*	SLE	2D HPLC-MS/MS (QqQ)	HILIC dimension<br>YMC-Pack Diol (*100 x 2.1mm ID, 5 μm*) with a Diol-NP guard column (*10 mm ² x 2.1mm, 5 μm*)<br>A: H_2O<br>B: ACN/ H_2O (90:10 *v/v*)<br>Both with 5 mM NH_4HCO_2 and 0.1% HAC	26	0.2-50	0.05-0-06 At<br>0.08-0.12 Sc	0.16-0.20 At<br>0.27-0.40 Sc	2-6 At<br>3-11 Sc	75-119 At<br>72-116 Sc	[39]
Sc + L-hyoscyamine + other (spartein)<br>*Porcine muscle*<br>*Egg and milk*	Modified QuEChERS	HPLC-MS/MS (QqQ)	C18<br>Phenomenex Kinetex EVO C18 (*150 x 4.6 mm ID, 5 μm*)<br>A: H_2O, B: MeOH, both with 0.1 % FA and 10 mM NH_4HCO_2	8	2-30	1 Sc<br>0.8 L-Hyoscy.	5 Sc<br>2 L-Hyoscy.	1-5 Sc<br>1-8 L-Hyoscy.	93-99 At<br>75-99 Sc	[30]
At, Sc + 2 Tas<br>*Herbal teas, herbal supplements*	SLE	UHPLC-MS/MS (QqQ)	C18<br>C18 EVO Kinetex (*100 x 2.1 mm ID, 2.6 μm)*<br>A: H_2O, B: ACN, both with 0.2% FA	15	0.5-5	N.S.	N.S	0-13 TAs<br>0-4 At<br>2-13 Sc	83-107 TAs<br>105-107 At<br>83-103 Sc	[25]

At, Sc + 4 TAs + others (ergot alkaloids) *Bread (wheat, multi-grain, rye, wheat-rye)*	SLE	UHPLC-MS/MS (QqQ)	C18 Waters Acquity BEH (*160 x 2.1 mm ID, 1.7 μm*), A: Ammonium carbonate (10 mM, pH 9), B: ACN	15	1-100	0.2-0.3 TAs 0.3 At 0.3 Sc	0.5-1 TAs 1.0 At 0.8 Sc	6-12 TAs 6 At 12 Sc	73-94 TAs 83 At 73 Sc	[23]
At, Sc *Honey*	SLE	UHPLC-MS/MS (QqQ)	HILIC coupled C18 Kinetex HILIC core–shell column (*100 x 2.1 mm ID, 1.7 μm)* and HILIC SecurityGuard ULTRA guard cartridge (2.1 mm) A: H_2O, B: ACN/water (95:5, *v/v*), both with 0.1% FA and 5 mM NH_4HCO_2	20	0.01-4	0.002 At 0.003 Sc	0.01 At 0.01 Sc	N.S	87-103 At 87-106 Sc	[33]
At, Sc + 9 TAs *Honey*	Modified QuEChERS	HPLC-HRMS (Orbitrap)	HILIC ACE HILIC-A (*250 x 4.6 mm ID, 3 μm*) A: H_2O with 0.1% FA and 5 mM NH_4HCO_2, B: MeOH	18	5-100*	N.S	20 At 20 Sc	0.1-20 TAs 1-18 At 0.1-20 Sc	71-120 TAs 85-103 At 116-120 Sc	[32]
At, Sc *Wheat, corn, rice, oat and millet flours, mixed cereals flours, infant cereals, cereal-based products*	QuEChERS	HPLC-MS/MS QqQ	C18 Acquity UPLC BEH C18 (*100 x 2.1 mm ID, 1.7 μm*), VanGuard BEH C18 pre-column (*2.1 x 5 mm ID, 1.7 μm*) A: H_2O with 1.5 mL/L FA and 10 mM NH_4HCO_2, B: MeOH with 0.5 mL/L FA	6	N.S	N.S	0.5	2-5 At 1-6 Sc	95-107 At 87-118 Sc	[19]
At, Sc *Gluten-Free Grains and Flours*	SLE-SPE	HPLC-MS/MS (QqQ)	C18 C18 Kromaphase 100 (*150 x 2.0 mm ID, 3.5 μm particle size*), C18 Kromaphase guard (*10 mm × 4.0 mm I.D, 5 μm particle size*) A: H_2O, B: ACN, Both with 0.1% FA	15	1-500	0.04-0.5 At 0.2-1.3 Sc	0.1-1.5 At 0.7-4.4 Sc	4-13 At 6-19 Sc	93-105 At 93-96 Sc	[14]

At, Sc + 19 TAs + others (pirrolizidine alkaloids) Sorghum, oregano, herbal tea	SLE	UHPLC-MS/MS (QTRAP)	C18 Luna Omega C18 (150 × 2.1 mm I.D, 1.6 µm) A: H2O, B: MeOH, both with 0.2 % FA and 2 mM NH4HCO2	22	0.5-1000	N.S	0.5–10 TAs<br>0.5-2 At<br>0.5-2 Sc	1-19 Tas<br>2-11 At<br>1-9 Sc	78-115 TAs<br>98-111 At<br>84-110 Sc	[38]
At, Sc Spinach-based products	QuEChERS-dSPE	UHPLC-MS/MS (QqQ)	C18 Zorbax Eclipse Plus C18 (50 x 2.1 mm ID, 1.8 µm) A: H2O, B: MeOH, both with 0.1% FA and 5 mM NH4HCO2	5	0.025-25	N.S	0.014-0.018 At<br>0.015-0.018 Sc	0.3-4 At<br>0.2-5.2 Sc	94-103 At<br>91-98 Sc	[29]
At, Sc Thyme, basil and coriander	SLE-SPE	HPLC-MS/MS (QqQ)	C18 C18 Kromaphase 100 (150 x 2.0 mm ID, 3.5 µm particle size), C18 Kromaphase guard (10 mm × 4.0 mm I.D, 5 µm particle size) A: H2O, B: ACN, Both with 0.1% FA	15	0.005-0.2	0.8-2.1 At<br>2.6-6.8 Sc	1.2-2.2At<br>4-7.2 Sc	3-8 At<br>2-14 Sc	87-92 At<br>70-92 Sc	[28]
At, Sc + others (mycotoxins, plant growth regulators, pesticides) *Wheat, barley, rice, oats, spelt, rye*	SLE	2D-LC-MS/MS (QqQ)	1st dimension - HILIC Column YMC-Pack Diol-NP (*100 x 2.1 mm ID, 5 µm particle size*), guard column cartridge (*10 × 2.1 mm ID, 5 µm*) Trap - ZORBAX SB-C8 (*12 × 4.6 mm ID, 5 µm*) 2nd dimension - Raptor FluoroPhenyl (*50 × 2.1 mm ID, 2.7 µm*), Raptor Biphenyl (*50 × 2.1 mm ID, 2.7 µm*), FluoroPhenyl guard column (*5 × 2.1 mm ID, 2.7 µm*), 1st dimension - A: H_2O, B: ACN/water (90:10 *v/v*), both with 0.1% FA and 10 mM NH_4HCO_2	25	5-200	N.S.	5 At<br>5 Sc	4-12 At<br>3-9 Sc	103 At<br>105 Sc	[42]

	2nd dimension - A: H_2O, B: MeOH, both with 0.001% FA and 5 mM NH_4HCO_2		

Abbreviations: ACN: acetonitrile. At: Atropine. dSPE: dispersive solid-phase extraction. EtOH: Ethanol. FA: formic acid. HAC: Acetic acid. HILIC: Hydrophilic interaction chromatography. HPLC-HRMS: high-performance liquid chromatography-high resolution mass spectrometry. HPLC-MS/MS: high-performance liquid chromatography–tandem mass spectrometry. LC: liquid chromatography. L-Hyoscy.:L-Hyoscyamine. MeOH: methanol. MDL: method detection limit. MQL: method quantification limit. MS: Mass spectrometry. N.S: Not shown. Q: single quadrupole. QqQ: triple quadrupole. QTRAP: quadrupole-ion trap. QuEChERS: Quick, Easy, Cheap, Effective, Rugged & Safe. RSD: Relative Standard Deviation. Sc: Scopolamine. SLE: solid-liquid extraction. SPE: Solid-phase extraction. TAs: Tropane alkaloids. UHPLC-MS/MS: ultra-high-performance liquid chromatography–tandem mass spectrometry. 2D: Two-dimensional. *Expressed in μg/L.

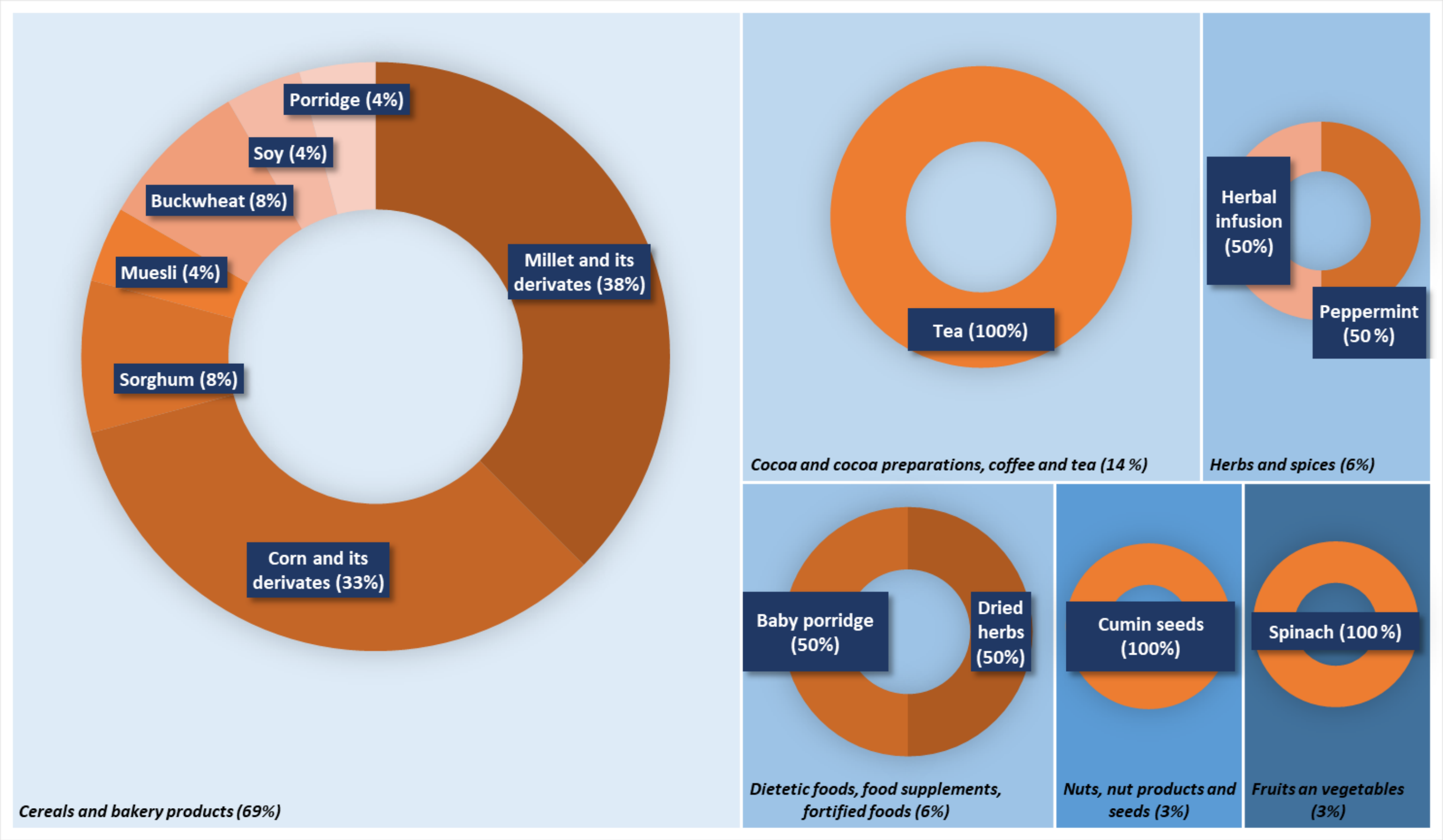

Figure S1. RASFF notifications of TAs by food category and food type (from 2015 to 2021).
